# Ways Forward for Tolerance-Inducing Cellular Therapies- an AFACTT Perspective

**DOI:** 10.3389/fimmu.2019.00181

**Published:** 2019-02-22

**Authors:** Anja ten Brinke, Marc Martinez-Llordella, Nathalie Cools, Catharien M. U. Hilkens, S. Marieke van Ham, Birgit Sawitzki, Edward K. Geissler, Giovanna Lombardi, Piotr Trzonkowski, Eva Martinez-Caceres

**Affiliations:** ^1^Department of Immunopathology, Sanquin Research, Amsterdam, Netherlands; ^2^Landsteiner Laboratory, Amsterdam UMC, University of Amsterdam, Amsterdam, Netherlands; ^3^Department of Inflammation Biology, MRC Centre for Transplantation, School of Immunology and Microbial Sciences, Institute of Liver Studies, King's College London, London, United Kingdom; ^4^Laboratory of Experimental Hematology, Faculty of Medicine and Health Sciences, Vaccine and Infectious Disease Institute (Vaxinfectio), University of Antwerp, Antwerp, Belgium; ^5^Institute of Cellular Medicine, Newcastle University, Newcastle upon Tyne, United Kingdom; ^6^Swammerdam Institute for Life Sciences, University of Amsterdam, Amsterdam, Netherlands; ^7^Charité-Universitaetsmedizin Berlin, Berlin Institute of Health, Institute for Medical Immunology, Humboldt-Universitaet zu Berlin, Berlin, Germany; ^8^Section of Experimental Surgery, Department of Surgery, University Hospital Regensburg, University of Regensburg, Regensburg, Germany; ^9^Division of Transplantation Immunology and Mucosal Biology, MRC Centre for Transplantation, Guy's Hospital, King's College London, London, United Kingdom; ^10^Department of Clinical Immunology and Transplantology, Medical University of Gdansk, Gdansk, Poland; ^11^Division of Immunology, Germans Trias i Pujol University Hospital, LCMN, IGTP, Badalona, Spain; ^12^Department of Cellular Biology, Physiology and Immunology, Universitat Autònoma de Barcelona, Cerdanyola del Vallès, Spain

**Keywords:** tolerance, dendritic cells, regulatory T cells, autoimmunity, transplantation, cell therapy, clinic, immunomonitoring

## Abstract

Clinical studies with cellular therapies using tolerance-inducing cells, such as tolerogenic antigen-presenting cells (tolAPC) and regulatory T cells (Treg) for the prevention of transplant rejection and the treatment of autoimmune diseases have been expanding the last decade. In this perspective, we will summarize the current perspectives of the clinical application of both tolAPC and Treg, and will address future directions and the importance of immunomonitoring in clinical studies that will result in progress in the field.

## Introduction

The number of patients with autoimmune diseases, severe allergies and recipients of organ or stem cell transplants is increasing worldwide. Currently, many of these patients require lifelong administration of immunomodulatory drugs, which cause generalized immunosuppression and hereby only partially alleviate the symptoms but do not cure the disease. Besides these drugs are inevitably associated with a risk of immediate or late-occurring severe adverse effects (e.g., life-threatening infections, cancer). Targeting the fundamental cause of autoimmunity, i.e., loss of tolerance to self-antigens, or inhibiting induction or execution of undesired immunity in transplantation and allergy will provide the next steps forward to avoid general immunosuppression. Accumulating knowledge on mechanisms of immune activation and tolerance has led to the development of tolerance-inducing cellular therapies with regulatory T cells (Treg) and tolerogenic antigen presenting cells (tolAPC), such as tolerogenic dendritic cells (tolDC) and regulatory macrophages (Mreg) [as reviewed in (1, 2)], with the specific objective to restrain unwanted immune reactions long-term.

The development of cell-based therapies is clinically attractive for many reasons, not in the least through their potential of being of low-toxicity, to simultaneously control many different inflammatory cells and induction of antigen-specific immunity. Since immunological tolerance is a self-reinforcing state (3), the therapeutic effects of cell therapy are expected to outlast the lifespan of the therapeutic cells themselves, opening the possibility of curative treatments. Production costs for these tolerogenic cell products range from 10,000 to 40,000 Euro depending on the therapeutic cell product and production site, which is relatively low considering that few injections of cells may be sufficient to induce long-lasting tolerance.

In recognition of the potential of tolerance inducing cell-based therapies and to join forces in the ongoing efforts in the field, A FACTT (Action to Focus and Accelerate Cell Based Tolerance-inducing Therapies (CTT) was initiated through EU COST Action Funding. Our goal was to initiate a network that would coordinate European CTT efforts to minimize overlap and maximize comparison of the diverse approaches across Europe. Now, looking back at 4 years of very active network interactions, we have evaluated our combined current stance in the field and defined avenues to support future directions.

## Tolerogenic Therapy With Antigen Presenting Cells in Clinical Practice

Over the past 20 years extensive experimental research has been invested in the generation and characterization of tolAPC, including tolDC and Mreg, with the aim to restore tolerance in autoimmune diseases (4–10) and transplant rejection (5, 11–14). To date, clinical trials exploring the safety, feasibility and efficacy of different types of tolAPC are a reality [reviewed in (1) and Table 1], and have confirmed so far that tolAPC therapy is safe, with no relevant side effects, and is well-tolerated by patients. Hence, to advance tolerogenic therapy with antigen-presenting cells (APC), we should stand on the shoulders of these pioneers and address remaining challenges, such as the optimal dose, injection route, frequency of administration, antigen-specificity, and the related issue of suitable biomarkers of cell therapy-induced reduction of general inflammatory state and induction of tolerance, in the design of the next-generation clinical trials.

**Table 1:** Completed or ongoing trials with tolAPC in autoimmunity and transplantation.

**Study ID**	**Phase**	**Cell product**	**Indication**	**Dosing scheme**	**tolDC per dose**	**Route of administration**	**Outcome**	**Center**	**References**
NCT00445913	I	Antisense ODN targeting CD40/CD80/CD86 tolDC	T1D	4 injections, bi-weekly	1 × 10^7^	Intradermal	-No adverse effects (AE) -Increase of B220+CD11c+ B cells -Evidence for C-peptide reactivation	Pittsburgh/USA	(15)
NCT02354911	II	Antisense ODN targeting CD40/CD80/CD86 tolDC	Recent onset T1D	4 injections, bi-weekly	1 × 10^7^	Intradermal	Not Recruiting	DiaVacs Inc. Pittsburgh/USA	
NTR5542	I	VitD3 and Dex tolDC pulsed with proinsulin peptide	T1D	2 injections, 4 weeks apart	Dose-escalation: 5, 10, or 20 × 10^6^	Intradermal	Finished	Leiden/NL	
Rheumavax	I	NF-kB inhibitor Bay 11-7082 tolDC pulsed with 4 citrullinated peptides	HLA-risk positive RA (minimal disease activity)	Single injection	Low dose 0.5-1 × 10^6^ High dose 2-4.5 × 10^6^	Intradermal	-Grade I AE. -Decrease DAS28 -Decrease eff T cells, ratio Teff/Treg -Decrease pro-inflammatory cytokines and chemokines	Brisbane/AUS	(16)
NCT01352858 (AutoDECRA)	I	Dex and VitD3 tolDC pulsed with autologous synovial fluid	Inflammatory Arthritis	Single injection	Dose-escalation: 1 × 10^6^ 3 × 10^6^ 10 × 10^6^	Intraarticular	-Safe, feasible, acceptable -Knee symptomse stabilized in 2 patients receiving the highest doses	Newcastle upon Tyne/UK	(17)
CreaVax-RA CRiS KCT0000035	I	tolDC pulsed with recombinant PAD4, RA33, citrullinated, fillagrin and vimentin	RA	5 injections	Low dose 0.5 × 10^7^ High dose 1.5 × 10^7^	Not indicated	-Treatment well tolerated -Antigen –specific autoantibodies decreased in 5/9 positive patients	Seoul/KOR	(18)
NCT03337165 (TolDCfoRA)	I	Dex and IFN-α tolDC	RA	Single injection	Dose-escalation: 1, 3, 5, 8, 10 × 10^6^	Intraarticular	Recruiting	Novosibirk/RUS	
NCT02283671	I	Dex tolDC pulsed with relevant disease peptides	MS and Neuro-myelitis optica	3 injections, bi-weekly	Not indicated	Intravenous	Recruiting	Barcelona/ES	
NCT02618902	I/IIa	VitD3 tolDC pulsed with myelin-derived peptides	Active MS patients	6 injections, 4 bi-weekly and 2 monthly	Dose-escalation: 5 × 10^6^ 10 × 10^6^ 15 × 10^6^	Intradermal	Recruiting	Antwerp/BE	
NCT02903537 (Tolervit-MS)	I/IIa	VitD3 tolDC pulsed with myelin-derived peptides	Active MS patients	6 injections, 4 bi-weekly and 2 monthly	Dose-escalation: 5 × 10^6^ 10 × 10^6^ 15 × 10^6^	Intranodal	Recruiting	Badalona, Pamplona/ES	
	I	Dex and VitA tolDC	Refractory Crohn's disease	Single injection or 3 injections bi-weekly	Dose-escalation: 2 × 10^6^ 5 × 10^6^ 10 × 10^6^	Intra-peritoneal	-No AE (3 patients withdrew due to worsening of symptoms) -Clinical improvement in 3 patients -Increase of Treg and decrease of IFN-⋎ levels	Barcelona/ES	(19)
NCT02622763	I	Dex tolDC	Crohn's disease	Not listed	10 × 10^6^ 100 × 10^6^	Intralesional	Recruiting	Barcelona/ES	
NCT02252055 (ONEatDC)	I/II	Low-dose GM-CSF-recipient tolDC	Kidney Tx from living donor	Single injection, 1 day before Tx	1 × 10^6^/kg bw	Intravenous		Nantes/FR	(20)
NCT03726307	I	VitD3 and IL-10 donor tolDC (Dcreg)	Kidney Tx from living donor	Single injection, 7 days before Tx	Dose - escalation: 0,5 ± 0,1 × 10^6^/kg bw, 1,2 ± 0,2 × 10^6^/kg bw, 2,5 to 5 × 10^6^/kg bw	Intravenous	Not yet recruiting	Pittsburgh/USA	
NCT03164265	I	VitD3 and IL-10 donor tolDC (Dcreg)	Liver Tx	Single injection, 7 days before Tx	Not described	Intravenous	Enrolling by invitation	Pittsburgh/USA	
TAIC-I	I/II	Cell product containing donor Mreg	Kidney Tx from deceased donor	Single injection, 5 days after Tx	0.5–7.5 × 10^6^/kg bw	Intravenous	Safe	Regensburg/DE	(21)
TAIC-II	I	Cell product containing donor Mreg	Kidney Tx from living donor	Single injection, 5 days before Tx	1.7–10.4 × 10^7^/kg bw	Intravenous	-Safe -No acute or delayed AE were associated with the infusion. −3 of the 5 patients were able to tolerate low-dose tacrolimus monothera- py and one patient was withdrawn from all immunosuppression for over 8 months	Regensburg/DE	(22)
NCT02085629	I/II	Donor derived Mreg	Kidney Tx from living donor	Single injection, 7 days before Tx	2.5–7.5 × 10^6^/kg bw	Intravenous	Completed, no results yet	Regensburg/DE	(20)

*This table is based on information deposited on www.clinicaltrials.gov, www.clinicaltrialsregister.eu and/or indicated references. AE, adverse event; RA, rheumatoid arthritis; T1D, type 1 diabetes; Tx, transplantation; Dex, dexamethasone; MS, multiple sclerosis; /kg bw, per kg body weight; vitD3, vitamin D3*.

### Phenotypic and Functional Identification of *in vitro* Generated TolAPC

Both tolDC and Mreg can be generated *in vitro* starting from CD14^+^ monocytes and share some phenotypic and functional characteristics. Indeed, both tolAPC types express low to intermediate levels of T-cell costimulatory molecules, and secrete low amounts of pro-inflammatory cytokines, indicative of a partially matured APC. Similarly, immature DC (iDC) display minimal expression of costimulatory molecules and little secretion of inflammatory cytokines, demonstrating potential optimal requirements for tolerance induction *in vivo* (23, 24). However, iDC are unstable and may differentiate into immunogenic DC under inflammatory conditions (25, 26). This invalidates their putative use as therapeutic products for tolerance induction. Therefore, different strategies to generate stable tolAPC have been explored, including treatment with pharmacological agents or cocktails of immunomodulatory cytokines, genetic engineering, and exposure to apoptotic cells (9, 27, 28). Most of these *in vitro* conditioning regimens aim at stabilizing a semi-mature state of tolDC, maintaining the capacity to induce immune hyporesponsiveness of T cells, even in presence of powerful pro-inflammatory signals.

Importantly, tolAPC inhibit T cell proliferation, albeit through different immunosuppressive mechanisms depending on the approach used to generate tolAPC *in vitro*. Induction of peripheral T cell anergy and apoptosis (29), attenuation of effector and memory T cell responses and the generation and activation of Treg populations (30, 31) result in part from presentation of low levels of antigen in the absence of costimulation; these are typical mechanisms attributed to a variety of tolerogenic subtypes (10, 32, 33). Additionally, tolerogenic DC may express various inhibitory receptors such as programmed death-ligand (PD-L)1, PD-L2 (34), immunoglobulin-like transcripts (ILT) (35), FasL (36, 37), and TRAIL (38). Secretion of anti-inflammatory cytokines such as IL-10 (39, 40) and TGF-β (41, 42), as well as reduced expression of pro-inflammatory cytokines, also may contribute to tolerance induction. A study comparing tolDC generated in presence of dexamethasone and rapamycin demonstrated that while both tolDC subsets were able to impair T cell proliferation, rapamycin-treated tolDC have a mature phenotype and are not able to produce IL-10 upon stimulation with LPS, as opposed to vitamin D_3_- or dexamethasone-treated tolDC (27). Whereas, it was demonstrated that rapamycin-treated tolDC induce Treg (27), vitD_3_-treated tolDC induce T-cell hyporesponsiveness and antigen-specific Treg (7, 43). Moreover, DC-10 induce Tr1 cells (44), while autologous tolDC have a weak capacity to stimulate allogeneic T cells and suppress T-cell proliferation and IFN-γ production (45). Mreg have been shown to convert allogeneic CD4^+^ T cells to IL-10-producing, TIGIT^+^, FoxP3^+^-induced Treg (46). Variations in the process to generate tolAPC may initiate regulation through distinctive mechanisms, making it difficult to compare these different types of tolAPC. Therefore, efforts have been made to find common features unique for tolerance-inducing cells (47). For example, since tolDC conditioned using vitamin D_3_ and dexamethasone exhibit high cell surface expression of TLR2 (48) or CD52 (49), such markers might be considered to assess the quality and stability of tolDC in future cell-based clinical trial protocols. In addition, it was demonstrated that the expression of single immunoglobulin IL-1-related receptor has a role in maintaining low levels of costimulatory molecules and in regulation of Treg expansion (50). Others demonstrated that C-lectin receptor CLEC-2 upregulation by DC is associated with Treg induction (51). So far, however, gene expression studies comparing different tolDC and Mreg protocols have not been able to identify common biomarkers of tolerance induction [reviewed by (52, 53) and (54)].

The difficulty in comparing characteristics of different clinical tolAPC suggests the need for a uniform set of metrics for their description, including full characterization of (at least) the immune phenotype and their functional activity (potency). Hence, a better identification of the characteristics that identify the tolerance-inducing properties of tolAPC, irrespective of the conditioning regimen, would be valuable for safe cell therapy delivery into patients. Joint efforts in translating tolAPC into the clinic by harmonizing protocols and defining functional quality parameters have been initiated (1, 55). A Minimum Information Model on Antigen-presenting cells (MITAP) has been defined to harmonize reporting on tolAPC therapy to ultimately allow the uncovering of commonalities between tolAPC and to define common quality control biomarkers and potency assays for the various tolAPC products for clinical use (55). Likewise, using similar immunomonitoring protocols in different clinical trials could help to better understand the *in vivo* mechanism of action of these cells (56).

### Antigen Specificity of TolAPC-Based Immunomodulation

Targeted regulation of antigen-specific T cell responses would avoid generalized immunosuppression as observed with immunosuppressive drugs and monoclonal antibodies currently in use in the clinics and may thus overcome occurrence of impaired immune-surveillance leading to infections or development of malignancies. *Ex vivo* generated tolAPC have the potential to therapeutically induce, enhance, or restore antigen-specific tolerance. Indeed, after loading these cells with exogenous or endogenous antigens, one major advantage is their capability to act in an antigen-specific manner.

A number of *in vivo* studies demonstrate that antigen loading of tolAPC is indispensable to reach efficient clinical responsiveness following tolAPC therapy. For instance, a beneficial effect of vitamin D_3_-tolDC loaded with MOG_40−55_ peptide was demonstrated in experimental autoimmune encephalomyelitis (EAE), whereas no clear beneficial effect on the clinical score of EAE mice was found when mice were treated with vitamin D_3_- tolDC not loaded with myelin peptides (57, 58). Similar findings have been demonstrated in other animal models of autoimmune diseases, including collagen-induced arthritis and autoimmune thyroiditis (59–61). Altogether, these findings suggest that selection of the target self-antigen is critical for disease-specific tolerance induction *in vivo*. Suitable disease-associated self-antigens responsible for T cell priming have been identified for T1D and multiple sclerosis (MS). However, this is not the case for other autoimmune diseases, such as rheumatoid arthritis or Crohn's disease, for which specific disease-associated antigens are unknown or not tissue specific. Moreover, not all patients uniformly display the same set of self-antigens for a given disease. MS, for example, is associated with a range of self-antigens and auto-antibodies that are differentially expressed among patients and at different points during the disease (62). While the targetable autoreactive T cell populations may be limited to a select number of antigens at the onset of clinical disease, other “late antigen” or spread epitope autoreactive T cell populations may drive autoimmunity during progression of the disease. To overcome this hurdle, some groups have chosen to load the tolerance-inducing therapeutic cells with a broad pool of distinct, candidate disease-related peptides (63–65) (NCT02283671, NCT02618902, and NCT02903537).

In contrast to autoimmunity, transplant rejection is mediated by an undesired immune response against epitopes that differ between the transplanted donor graft and the recipient host, so-called allorecognition (66). Specifically, recipient T cells may initiate a strong immune response leading to transplant rejection in the absence of adequate immunosuppression. To avoid transplant rejection, the induction of tolerance to donor-specific antigens has been coined as a therapeutic target for decades. For this, both donor tolAPC and recipient tolAPC loaded with donor-specific antigens are being considered for development of cell-based immunotherapeutic protocols in the transplantation setting (67). However, whereas the clinical use of donor-derived tolAPC is only feasible in the context of living donor transplantation and entails a risk of sensitization (including development of allo-antibodies), the use of autologous, i.e., recipient-derived, tolAPC is a less risky approach to begin with. Indeed, the use of recipient autologous tolAPC is likely to be more feasible than that of donor-derived tolAPC, since cell products can be prepared from the peripheral blood of the recipient before transplantation, stored while the patient is on the waiting list, and loaded with donor-derived antigens (such as HLA peptides or donor cell lysates) at the time of transplantation. In the context of the ONE study, two trials using tolDC and Mreg are being performed in living-donor kidney transplant recipients (Table 1).

### Route of Administration of TolAPC

Although it is generally accepted that the route of administration is important for optimal tolAPC effector function, the best route of tolAPC administration is not known. To date, a variety of routes of administration have been used (see Table 1), including intradermal, intraperitoneal (19), intravenous and intra-articular (17). Different routes of administration may be required to allow tolDC to reach the relevant draining lymphoid tissue for T cell encounter or to end up at the site of inflammation. Especially since tolDC demonstrate a reduced expression of CCR7 and consequently a reduced (but not absent) ability to migrate in response to the CCR7 ligand CCL19 (68), the capacity of tolDC to reach the lymph nodes is a critical concern. While the migration of DC toward the lymph nodes increases following intradermal as compared to subcutaneous administration, only 2–4% of DC migrate to the draining lymph nodes after intradermal administration, but the situation may be different in patients with autoimmune diseases where monocyte-derived DC from MS patients have shown a significantly higher proportion of CCR7-expressing cells compared to healthy controls (69). Given these observations, and that in the setting of cancer vaccine development, DC injected into a lymphatic vessel showed a prolonged half-life as compared to DC injected intravenously (70, 71), direct intranodal injection of tolDC is being evaluated in a clinical setting (see Table 1).

As an alternative to lymph node targeting of tolAPC, tolAPC may also be introduced directly into the site of inflammation. Indeed, injection into the affected disease site (i.e., an inflamed joint) where the tolAPC could suppress auto-reactive effector T cell responses is logical. In this context, intra-articular injection of tolDC differentiated using dexamethasone and vitamin D_3_ and loaded with autologous synovial fluid in patients with rheumatoid arthritis was demonstrated to be safe and feasible. Hence, despite the fact that tolDC were directly injected at the site of inflammation, no adverse events were observed in most patients and hypertrophy, vascularity and synovitis were stable in all treated cohorts. Moreover, two patients receiving 10 million cells showed a decrease in synovitis score (17). Similarly, a phase I randomized clinical study currently evaluates the safety and efficacy of tolDC injected into the intestinal lesions in patients with refractory Crohn's disease (Table 1). In some conditions such as T1D, direct injection of cells in the inflammatory site, e.g., pancreas, might not be possible and require tolDC administration adjacent to the inflammation site. For the treatment of inflammatory diseases of the brain, the blood–brain barrier (BBB) may represent a major hurdle. Considering this potential problem, it was demonstrated that enhancing CCR5 expression in tolDC using mRNA electroporation endowed these cells with CCR5-driven migratory capacity. This enabled the cells to migrate to inflammatory sites, even when it required crossing of functional barriers such as the BBB (72). Similarly, introducing CCR7 expression in tolDC using the proposed approach of chemokine receptor mRNA electroporation could overcome the limited lymphoid homing capacity of tolDC. Indeed, DC transduced with lentiviral vectors coding for CCR7 and IL-10 genes were able to migrate to the lymph nodes and spleen, prolonging cardiac allograft survival in mice (73). However, there are still many unknowns and there is a clear clinical need to characterize the pharmacodynamics of tolDC in humans and relate this to clinical efficacy. Advances in cell imaging techniques, for example magnetic resonance imaging of 19F-labeled cells, have made it possible to address this question in future studies.

### TolAPC Therapy: What Does the Future Hold?

#### *In vivo* Targeting

While our knowledge of tolAPC biology has expanded greatly, and *in vitro* generated tolDC and Mreg are currently being used in various clinical trials (Table 1), clinical-grade manufacturing of tolAPC is still a time-consuming and expensive process. It requires cell precursors that need to be isolated from the patient's blood, modulated *ex vivo* and reintroduced into the patient. Direct antigen delivery to tolAPC *in vivo* may limit the workload and costs. Indeed, specific antigen-targeting of DC-restricted endocytic receptors (DEC-205) with monoclonal antibodies has been shown to induce antigen-specific T cell hyporesponsiveness in experimental models (74). Interestingly, a phase I clinical trial demonstrated that *in vivo* targeting of human DC could be achieved by antibodies against DEC205 with subsequent antigen presentation and robust humoral and cellular responses (75). *In vivo* targeting of DC with biomaterials such as liposomes, microparticles and nanoparticles is also a promising approach [as reviewed in (76–78)]. This is exemplified by the fact that liposomes loaded with NFkB inhibitors targeting APC *in situ*, suppress the cellular responsiveness to NF-kB and induce antigen-specific FoxP3^+^ regulatory T cells in an animal model of arthritis (79) and that administration of phosphatidylserine-rich liposomes loaded with disease-specific autoantigens lead to a beneficial effect in experimental models of T1D and MS (80, 81). Nevertheless, DC represent a heterogeneous cell population arising from bone marrow-restricted precursors identified in humans. While multiple subsets of DC have been found in the peripheral blood, lymphoid organs and tissues, most of the hallmark DC markers are promiscuously expressed making it difficult to unambiguously discriminate between DC subpopulations and specifically target those DC subpopulations that induce tolerance. Extensive phenotypic screening combined with gene expression profiling allows the identification of tolerance-inducing DC counterparts present *in vivo*. For instance, Gregori and co-workers identified a DC subset expressing HLA-DR^+^CD14^+^CD16^+^ that exhibits potent tolerogenic activity (44). In targeting only such tolerance-inducing DC cell type-specific targeting may emerge as another promising approach in DC-based immunotherapy.

#### Combination Therapy

Since a variety of often complementary mechanisms are involved in the maintenance of immune tolerance, a more complex therapeutic approach using combinations that target different pathways that contribute to induction and maintenance of tolerance may be required to fully control autoimmunity. For instance, combinations of tolAPC with disease-modifying treatments that reduce the frequency of disease-causing cells, e.g., alemtuzumab, should be explored as the latter therapy reduces the disease-causing cells to a number that may be more effectively controlled by antigen-specific tolerance-inducing strategies, such as tolDCs. Alternatively, therapies like fingolimod, an antagonist of sphingosine−1-phosphate receptor which retains naïve and central memory T cells in the lymph nodes, are promising as co-medication with tolAPC as it could increase the number of tolDC-T cell interactions in the lymph node thereby facilitating Treg priming and consequently the efficacy of tolDC-based strategies. Also in the context of solid organ transplantation, tolDC therapy could be improved by the addition of a complementary treatment. For instance, the combination of adoptive transfer of tolDC and CTLA-4 Ig, a fusion protein that blocks B7-CD28 costimulation, resulted in extended survival of MHC-mismatched heart allografts in mice (82, 83), while the pancreatic islet allograft survival improved by combination of autologous tolDC and CD3 targeting antibodies (84). With the recent data on the important role of other coinhibitory molecules for T cell-mediated inflammation such as CD96 the portfolio of combination therapies might increase in the next years (85). However, since antigens can easily trigger immunity instead of tolerance, a primary concern remains the safety of combining two immune-modulatory vaccination strategies in autoimmune diseases and in the prevention of transplant rejection. Although one can envisage that concomitant use of immunosuppressive therapies might synergistically reduce the risk to unexpectedly worsen antigen-specific reactions (86), any novel manipulation of the immune system may involve an unpredictable risk. Furthermore, combination therapy may introduce confounding factors inducing additive, synergistic or antagonistic effects complicating the evaluation of the precise mode of action.

### Conclusion

Several protocols to generate and administer tolAPC have been tested in phase I clinical trials with highly encouraging results from a safety point of view and in terms of adverse effects (Table 1). Further phase I/II studies are under way in Crohn's disease, T1D, rheumatoid arthritis, MS and kidney transplantation (Table 1). However, for the success of future tolAPC trials, there is great need to define the optimal vaccination protocol; to ensure optimal *in vivo*-acting of the tolAPC, future trials may require changes in administration route, dose or could demand repeated tolAPC administration. Furthermore, the identification of a common set of tolerogenic markers would enable optimized comparison of tolAPC products and their tolerance-inducing potential and provide an improved understanding of how these cells modulate the T cell response both locally and systemically. It would be of great help to analyze critical pathways contributing to programming and function of tolAPC. Ultimately, this may set the stage for new approaches improving the therapeutic potential of tolAPC for the future.

## CD4^+^ Regulatory T Cells (Treg)

CD4^+^ Treg are recognized as a dominant cell population responsible for induction and maintenance of immune tolerance. They may be generated either in the thymus as natural regulatory T cells (nTreg or tTreg) or in the periphery as induced regulatory T cells (iTreg). Both subsets can induce tolerance toward auto- and alloantigens utilizing a variety of mechanisms including cell-to-cell contacts, secretion of immunosuppressive cytokines and inhibitory molecules (e.g., adenosine or prostaglandin E), local depletion of IL-2, or through killing of other cells (87). Treg actively traffic to inflammatory sites and the suppressive activity is usually localized without a significant impact on the general immunity. Since their more precise identification 2–3 decades ago in the mouse, and more recently in the human, steady advances in understanding Treg biology have eventually provided sufficient knowledge to culture, manipulate and expand the cells *in vitro* under Good Manufacturing Practice (GMP) conditions for therapeutic purposes. Indeed, Treg have become a promising cellular drug that can potentially be used to control disease-causing immune responses.

### Treg in Clinical Practice

While the application of Treg for the treatment of autoimmune diseases is currently under intense investigation, Treg were first used in the clinic to treat patients with graft vs. host disease (GvHD) after hematopoietic stem cell transplantation (HSCT) (88) (Table 2). Results from the clinical trials in GvHD with polyclonal expanded Treg have suggested that altogether these cells are safe, but there is some concern about the occurrence of mild to moderate infections, and it still is unclear whether Treg treatment could promote cancer (92, 94). The latter problem has been reported in only one trial to date, however it was concluded that the tumor was present before the therapy with Treg was applied (94). The safety and feasibility of adoptive transfer of *ex vivo* expanded Treg was further confirmed in T1D patients (2), which has driven the application of Treg therapy to clinical trials in other autoimmune conditions such as MS, autoimmune hepatitis, systemic lupus erythematosus, Crohn's disease, and autoimmune uveitis (102) (Table 2). Another clinical trial was recently published where polyclonal Treg were injected into T1D patients; results from this trial confirm the safety of this type of therapy and also show for the first time, by deuterium labeling of the Treg, that some of the injected Treg can be detected for up to 1 year after infusion (103).

**Table 2:** Completed or ongoing trials with Treg in autoimmunity and transplantation.

**Study ID**	**Phase**	**Cell product**	**Indication**	**Dosing scheme**	**Tregs per dose**	**Outcome**	**Center**	**References**
**HSCT**								
NKEBN/458-310/2008	I	Expanded polytTregs	GvHD treatment	Single injection or3 injections	1 × 10^5^/kg bw 3 × 10^6^cells/kg bw	-Safe -Reduced immunosuppression in chronic GVHD -Only transient improvement in acute GVHD	Gdansk/PL	(88)
NCT00602693	I	Expanded CB polytTregs	GvHD prophylaxis	Single injection	Dose-escalation: 1, 3, 10, 30, 30+30, 100, 300, 1,000 and 3,000 × 10^5^/kg bw	-Safe -Increased incidence of infections -Reduced incidence of acute GvHD/GvL effect	Minnesota/USA	(89, 90)
01/08	I	Fresh polytTregs	GvHD prophylaxis	Single injection	Dose-escalation: 0.5 × 10^6^ Tcons/kg bw with 2 × 10^6^ Tregs/kg bw 1 × 10^6^ Tcons/kg bw with 2 × 10^6^/Tregs kg bw −2 × 10^6^ Tcons/kg bw with 4 × 10^6^ Tregs/kg bw	-Safe -Reduced number of leukemia relapses -Reduced incidence of GVHD	Perugia/IT	(91, 92)
Treg002 EudraCT: 2012-002685-12	I	Fresh polytTregs	GvHD prophylaxis	Single injection	up to 5 × 10^6^/kg bw	Safe	Regensburg/DE	(93)
EK 206082008	I	Expanded polytTregs	GvHD treatment	Single or 2 injections	0.97–4.45 × 10^6^/kg bw	-Two cases of tumors -Stable chronic GvHD	Dresden/DE	(94)
ALT-TEN	I	Tr1 (*IL-10 DLI* or *DC-10 DLI*)	GvHD prophylaxis	Single injection	Dose-escalation: 1 × 10^5^, 3 × 10^5^ and 1 × 10^6^, 3 × 10^6^ CD3^+^ T cells/kg bw	-Safe -Long-term free-disease survival in 4 patients	Milan/IT	(95)
NCT02749084	I/II	Multiple Treg DLI	Severe Refractory Chronic GvHD prophylaxis	3 injections	Dose-escalation: 1.7 × 10^5^, 3.3 × 10^5^ and 6.6 × 10^5^/kg bw per injection	Recruiting	Bologna/IT	
NCT02991898	II	Fresh CB polyTregs with IL-2	aGvHD prophylaxis after CB Tx	Single injection	No data	Suspended	Minnesota/USA	
NCT01911039	I	polyTregs	Steroid Dependent/ Refractory Chronic GvHD treatment	Single injection	Dose-escalation: 1 × 10^5^, 5 × 10^5^, 1.5 × 10^6^ /kg bw	Unknown	Stanford/USA	
NCT02385019	I/II	Fresh donor polyTregs	Steroid-Refractory Chronic GvHD treatment	Single injection	Dose-escalation: 0.5 × 10^6^, 1.0 × 10^6^ and 2.0–3.0 × 10^6^/kg bw	Recruiting	Lisboa/PT	
NCT01937468	I	Fresh polyTregs with IL-2	Steroid -Refractory Chronic GvHD treatment	Unknown	Unknown	Active/not recruiting	Boston/USA	
NCT01903473	I	Fresh polyTregs with rapamycin	aGvHD and cGvHD treatment	Single injection	≥ 0.5 × 10^6^/kg bw	Unknown	Liege/BE	
EudraCT: 2012-000301-71	I	Fresh polyTregs with rapamycin	Steroid -Refractory Chronic GvHD treatment	Single injection	≥ 0.5 × 10^6^/kg bw	Recruiting	Liege/BE	
NCT01795573	I	Donor polyTregs expanded with recipient DC	aGvHD prophylaxis	Unknown	Unknown	Recruiting	Tampa/USA	
NCT01660607	I/II	Fresh polyTregs with Tconv	aGvHD prophylaxis	Single injection	initial doses will be 1 × 10^6^ Treg/kg bw to 3 × 10^6^ Tcon/kg bw (ratio 1:3)	Recruiting	Stanford/USA	
NCT02423915	I	Fucosylated fresh CB polyTregs	GvHD prophylaxis	Single injection	Dose-escalation: 1 × 10^6^/kg bw 1 × 10^7^/kg bw	Active/Not Recruiting	Houston/USA	
BMT Protocol 204 NCT01050764	I	Fresh allogeneic polyTregs with Tconv	GvHD prophylaxis	Single injection	Dose-escalation: 1 × 10^5^ Treg and 3 × 10^5^ T con/kg bw or 3 × 10^5^ Treg and 1 × 10^6^ Tcon/kg bw or 1 × 10^6^ Treg and 3 × 10^6^ Tcon kg/bw or 3 × 10^6^ Treg and 1 × 10^7^ Tcon/kg bw	Terminated, GvHD was within parameters to continue, but the study was terminated due to poor outcomes prior to sufficient accrual to set the MTD, even at the only dose tested (1 × 10^5^ Treg and 3 × 10^5^ T con/kg bw). Primary outcome result is null.	Stanford/USA	
NCT01634217	I	iTregs	GvHD prophylaxis	Unknown	Unknown	Active, not recruiting	Minnesota/USA	
**TRANSPLANTATION**								
NCT02129881	I/II	Expanded polytTregs	Living donor kidney Tx	Single injection	1–10 × 10^6^ /kg bw	Completed, no results yet	London, Oxford/UK	(20)
ONEnTreg13 NCT02371434 EudraCT: 2013-001294-24	I/II	Expanded polytTregs	Living donor kidney Tx	Single injection	Dose-escalation: 0.5 × 10^6^, 1 × 10^6^, and 3 × 10^6^/kg bw	Completed, no results yet	Berlin/DE	(20)
DART NCT02244801	I/II	Donor-Alloantigen-Reactive (dar) Tregs	Living donor kidney Tx	Single injection	Dose-escalation: 300 × 10^6^ darTreg or 900 × 10^6^darTreg	No longer recruiting	San Francisco/USA	(20)
NCT02091232	I/II	Belatacept-conditioned Tregs	Living donor kidney Tx	Unknown	Unknown	No longer recruiting	Boston/USA	(20)
ThRIL NCT02166177	I	Expanded polytTregs	Liver Tx	Single injection	low dose and high dose	Completed, no results yet	London/UK	
NCT02188719	I	Donor-Alloantigen-Reactive Tregs	Liver Tx	Single injection	Dose-escalation: 0 × 10^6^ darTreg or 50 × 10^6^ darTreg or 200 × 10^6^ darTreg or 800 × 10^6^ darTreg	Recruiting	San Francisco/USA	
NCT02088931	I	Expanded polytTregs	Subclinical rejection in kidney Tx	Single injection	200 × 10^6^	Open/Not recruiting	San Francisco/USA	
NCT02474199	I	Donor-Alloantigen-Reactive Tregs	CNI reduction in liver Tx	Single injection	300–500 × 10^6^ /kg bw	Recruiting	San Francisco/USA	
NCT01624077	I	Donor-antigen expanded Tregs	Liver Tx	Multiple injections at several intervals	1 × 10^6^ /kg bw per injection	Unknown	Nanjing/CHN	
NCT01446484	I	polytTregs	Living donor kidney Tx	Single injection sub-cuntaneous	2 × 10^8^ s.c.	Unknown	Moscow/RUS	
TRACT NCT02145325	I	Expanded polytTregs	Living donor kidney Tx	Unknown	Unknown	Active, Not recruiting	Chicago/USA	
NCT02711826	I/II	Donor-Alloantigen-Reactive Tregs vs. polytTregs	Subclinical rejection in kidney Tx	Single injection	400 ± 100 × 10^6^ darTregs	Recruiting	Birmingham, Los Angeles, San Francisco, Ann Arbor, Cleveland/USA	
**AUTOIMMUNITY**								
TregVAC ISRCTN06128462	I	Expanded polytTregs	Recent T1D	Single or 2 injections	Dose-escalation: 10 × 10^6^, 20 × 10^6^, or 30 × 10^6^/kg bw	-Completed -Safe -Reduced insulin consumption (insulin independence in 2 out of 12 patients) -Better stimulated C-peptide secretion profiles	Gdansk/PL	(96–98)
NCT01210664	I	Expanded polytTregs	T1D	Single injection	Dose-escalation: 0.05 × 10^8^, 0.4 × 10^8^, 3.2 × 10^8^, 26 × 10^8^	Completed/Safe	San Francisco/USA	(99)
CATS1	I/II	Ovalbumin-specific Tr1	Refractory Crohn's disease	Single injection	Dose-escalation: 1 × 10^6^, 10^7^, 10^8^, or 10^9^	-Safe -Clinical response in 40% of patients	Lille/FR	(100)
CATS29 EudraCT: 2014-001295-65 NCT02327221	II	Ovalbumin-specific Tr1	Refractory Crohn's disease	Single injection	1 × 10^6^/kg bw	Terminated/completed	Valbonne/FR Multicenter: AT, BE, FR, GE, IT, UK	
TregVAC2.0 EudraCT: 2014-004319-35	II	Expanded polytTregs combined with antiCD20 antibody	Recent T1D	2 injections, 3 months apart	30 × 10/kg bw per injection	Recruitment closed/Follow up in progress	Gdansk/PL	
TregSM EudraCT: 2014-004320-22	I	Expanded polytTregs	MS	Single injection: Cohort I – *intravenous* Cohort II - *intrathecal*	Up to 40 × 10^6^/kg bw	Recruiting	Gdansk/PL	
NCT02704338	I	Expanded polytTregs	Autoimmune hepatitis	Single injection	10-20 × 10^6^/kg bw	Not yet recruiting	Nanjing/CHN	
NCT02772679	II	Expanded polytTregs with IL2	Recent T1D	Single injection	3 or 20 × 10^6^/kg bw	Suspended	San Francisco/USA	
NCT02428309	II	Expanded polytTregs	Systemic lupus erythematosus	Single injection	Dose-escalation: 1 × 10^8^ or 4 × 10^8^ or 16 × 10^8^	Active/Not Recruiting	San Francisco/USA	
NCT02932826	I	Expanded third-party CB polyTregs	Recent T1D	Single injection	1–5 × 10^6^/kg bw	Recruiting	Hunan/CHN	
NCT03011021	I	Expanded third-party CB polyTregs and Liraglutide	Recent T1D	Single injection	1–5 × 10^6^/kg bw	Recruiting	Hunan/CHN	
T-Rex Study NCT02691247	II	Expanded polytTregs	Recent T1D	Single injection	low dose and high dose	Active/Not Recruiting	San Francisco, Aurora, New Haven, Gainesville, Miami, Indianapolis, Boston, Fargo, Kansas City, Portland, Sioux Falls, Nashville/USA	
**OTHER**								
NCT03101423	I	Donor polyTregs DLI	Beta Thalassemia Major	Unknown	Unknown	Active/Not Recruiting	Nanning/CHN	
NCT03241784	I	Donor polyTregs DLI	Amyotrophic Lateral Sclerosis (ALS)	8 injections *iv* in total concomitant with IL2 *sc*.: 4 injections over 2 months at early stage and 4 injections over 4 months at later stages	1 × 10^6^/kg bw per injection	completed	Houston/USA	(101)

*This table is an updated version of the table in (102) and is based on information deposited on www.clinicaltrials.gov, www.clinicaltrialsregister.eu and/or indicated references. Route of administration of the Tregs is intravenous, except where otherwise noted. AE, adverse event; T1D, type 1 diabetes; Tx, transplantation; MS, multiple sclerosis; /kg bw, per kg body weight; HSCT, hematopoetic stem cell transplantation; CB, cord blood; GvHD, graft vs. host disease*.

Treg therapy is now being applied as a “tolerogenic” therapy to reduce dependency on immunosuppressant drugs in patients receiving solid organ transplants. The idea behind this strategy is very similar to the application of Treg in autoimmune diseases, namely to tilt the balance toward Treg dominance over rejection-causing Teff cells. The first reports using adoptive transfer of Treg in kidney transplant patients have been recently published demonstrating the safety of this strategy in the context of solid organ transplantation (103, 104). Recently, clinical trials are being completed using different variations of Treg products (*The ONE Study* and ThRIL) (Table 2). *The ONE Study* includes Phase I clinical trials comparing the safety of different types of regulatory cells, including polyclonal and donor-reactive Treg in patients receiving kidney transplants (www.onestudy.org) (20). The ThRIL trial is a Phase I/IIa dose-escalation clinical trial in the setting of liver transplantation. Results from the ThRIL and the various *ONE Study* trials are currently being prepared for publication. The impact of Treg on the recipient immune system will be revealed only when the very detailed immunomonitoring is completed, which is a major objective of the described clinical trials (105–107).

Altogether, from the outcomes of the completed clinical trials so far, it can be concluded that adoptive transfer of Treg is safe and technically feasible (Table 2). Therefore, increasing efforts are currently focusing on clinical trials to test their therapeutic efficacy. Importantly, several lessons have been learned from recent experiences with Treg to improve future trial designs. For example, the clinical state of the patients has been shown to influence the function and properties of Treg, and therefore can condition the success of *ex vivo* cell product expansion (108). Furthermore, the specific expansion protocol can affect Treg function and specificity, and can improve tissue targeting and suppression capacity (108). Finally, the immune modulatory therapies received by patients at the time of Treg adoptive transfer can positively or negatively impact therapeutic outcome (109, 110).

### Treg Therapy: What Does the Future Hold?

#### Antigen-Specificity of Treg Therapy

Studies in preclinical models using murine Treg have demonstrated that specificity for either the auto or allo (transplantation) -antigens may offer an advantage for Treg function compared to polyclonal Treg (111). Adoptively transferred allospecific murine Treg generated by using either donor-derived APC or TCR transduction promote indefinite heart allograft survival, even in completely mismatched mouse strains (111). This strategy was subsequently applied to human Treg in a humanized mouse model, where Treg were generated in the presence of donor APC /DCs or B cells) and shown to be superior to polyclonal Treg in protecting from human skin graft rejection (112). More recently, by conferring specificity using a chimeric antigen receptor (CAR), human Treg transduced with a lentivirus encoding for HLA-A2-CAR were superior to polyclonal Treg in protecting HLA-A2^+^ human skin grafts (113–115). CAR constructs are now being developed to increase Treg stability and function.

Based on promising results with antigen-specific Treg in pre-clinical models, the use of alloantigen-specific Treg generated by culturing recipient Treg with donor-specific cells, either using activated donor-derived B cells (112) or donor-derived DCs is being tested in clinical trials (Table 2). Compared to the transplantation field where the antigens are known, generation of antigen-specific Treg in autoimmunity is more challenging because the inciting antigens are often not known (see also paragraph Antigen Specificity of tolAPC-Based Immunomodulation). New data suggest that regulatory and effector cell subsets are driven by different epitopes (116, 117). In addition, the auto-antigen triggering the autoimmune condition can change during disease progression due to epitope spreading and antigen-specific Treg may thus need to be tuned toward specific stages of disease.

#### Combination Therapy

Since adoptive transfer with Treg alone, particularly with a one-time infusion, may not be sufficient to control the immune response, combined or successive therapies are being tested. One approach is based on evidence that low doses of IL-2 can preferentially increase the endogenous pool of Treg; so far, low-dose IL-2 treatment has been safe in inflammatory conditions such as GvHD after HSCT (118). In a preclinical model it was demonstrated that IL-2/anti-IL-2 complexes not only promote Treg proliferation, they increase Treg survival and function while synergizing with calcineurin inhibitors to prolong graft survival (119). Recently, in a murine model of transplantation it was shown that by combining donor-specific Treg with the IL-2/anti-IL-2 complexes, a synergistic effect in extending skin transplant survival is observed (Ratnasothy et al., unpublished data). These results pave the way for the first clinical trial in liver transplant patients to combine Treg and low-dose IL-2 therapy (NCT02949492). More caution is being exercised regarding similar trials using low-dose IL-2 in autoimmune diseases, since in contrast to HSCT or solid organ transplantation, autoimmune patients are not lymphopenic and are likely to produce IL-2 themselves (98). However, in some conditions such as systemic lupus erythematosus an intrinsic defect in Tregs contributes to disease progression and here low-dose IL-2 therapy was shown to correct the defect (120, 121). Furthermore, in T1D settings low dose IL-2 treatment is currently being investigated (NCT02411253).

The positive effects of rapamycin on Treg *in vitro* or in a transplant setting (122–124) could not be effectively translated to the *in vivo* treatment of autoimmune syndromes. For example, while rapamycin administered to T1D patients preferentially increased Treg levels, pancreas function deteriorated due to islet toxicity (125). Nonetheless, the complex and specific pathogenesis of autoimmune syndromes may provide hints toward the design of new combined therapies. Tandemly targeting different effector mechanisms involved in particular syndromes with Treg therapy may improve outcomes. For example, an ongoing trial in early phase of T1D (EudraCT: 2014-004319-35) supports the idea of a synergistic approach by combining Treg administration with B cell depletion.

#### New Technical Advancements

It is now accepted that the optimal way to manufacture Treg for clinical use requires an efficient *ex vivo* expansion rate while maintaining purity and suppression potency before GMP product release. Two main Treg isolation strategies are currently being used in clinical trials to purify the starting Treg, immunomagnetic selection and flow cytometry cell sorting. The magnetic platform (CliniMACS CD4^+^CD25^+^ selection) provides a highly automated GMP-grade approach which is easy to standardize across centers. However, the resultant Treg product does generally contain a minor population of CD127^+^ cells that could jeopardize product purity after expansion. Addition of rapamycin to the culture media has helped to maintain Treg purity and function, without reducing the expansion rate too much (126). The second method, which uses a flow cytometry approach, results in a highly pure Treg population due to CD127^+^ cell depletion ability. However, this cell-sorting strategy entails more complex protocols and challenges to maintain GMP grade. With the appearance of new GMP-compatible cell sorters (Tyto MacQuant, Miltenyi or Influx, BD Biosciences), this sorting approach is likely to become the preferential Treg isolation method.

Although cell-sorting of Treg increases their initial purity, a search for an optimally stable final Treg products is critical therapeutically. It is well known that prolonged *in vitro* culture results in epigenetic changes in Treg—methylation of TSDR region of *foxp3* gene—which in-turn reduces suppressive ability (127). The use of anti-methylation agents in cultures to prevent epigenetic changes has failed due to culture viability issues. The addition of rapamycin to the expansion culture, as used in both *The ONE Study* and ThRIL, was proposed as a remedy to such changes as the drug preferentially expands Treg both *in vitro* and *in vivo* (122). However, proper choice of media, addition of autologous serum, limited time frame for the expansion and temperature decreases during *in vitro* culture to ≈33°C, all impact the suppressive capacity of final Treg products (128–130).

### Conclusion

Treg therapies are currently undergoing intense testing. An interest in therapeutic Treg preparations has resulted in several ongoing clinical trials in the transplantation setting, in autoimmune diseases, but now also in conditions such as beta thalassemia major and amyotrophic lateral sclerosis (101) (Table 2). We await new testing in the setting of hypersensitivity and cardiovascular disease (102), and anticipate that Treg therapy will be considered for any condition where there is evidence for an immune regulation imbalance.

## Timing of Tolerance-Inducing Cell Therapy

Timing of tolerance-inducing cell therapy in relation to the transplant or in the course of autoimmune disease development needs specific consideration. Clinical trials using Treg for GvHD indicate that Treg should be injected as early as possible, preferentially before disease onset (89, 91, 131). Early treatment is particularly important to achieve a high ratio between Treg and detrimental effector T cells, and thus prevent development of acute rejection or GvHD. In both HSCT and solid organ transplantation, tolerance-inducing cells are therefore mainly given around, just before or shortly after, the transplantation (see Tables 1, 2). Although early treatment is likely more effective, this approach encounters limitations such as increased immunosuppression doses and anti-CD25 treatment, which can interfere with the activity of infused cells. Thus, Tregs have also been infused at later time points e.g., 6 months after kidney transplantation in patients with biopsies showing evidence of inflammatory infiltrates to treat ongoing chronic rejection (103). It must also be considered that disease diagnosis timing is a factor in this respect. Although it is likely best to give the tolerance-inducing cell treatment as early as possible in disease development, it is currently not feasible in treating autoimmunity since a majority of autoimmune diseases start long before clinical symptoms and diagnosis, this brings in the added factor that the functional capacity of the attacked organ may already be irreparably damaged by the ongoing autoimmune process. Future insight into autoimmune disease development and early biomarkers will hopefully allow for earlier treatment with tolerance-inducing cell products.

## Regulations

The perspectives of tolerance-inducing cellular therapy depend also on recently introduced regulations. The majority of tested preparations in Europe are now classified as drugs under the 1394/2007 EU directive on advanced therapy medicinal products (ATMP). This significantly changes the legal path for their registration requirements, for manufacturing license and marketing authorization. While the idea of an ATMP in Europe is relatively nascent, and it is continuously evolving through public consultations with interested parties, cellular drugs have a distinct central paneuropean path for registration. While the Committee for Advanced Therapies (CAT) steers this process in Europe to optimize safety for patients, it would be useful to introduce wider rules allowing for introduction of the cells as a routine treatment. To accelerate the whole process, acknowledgment of flexible new types of manufacturing cGMP equipment and reagents could open the way to more widespread ATMP use. Furthermore, measures to reduce manufacturing costs would lessen this major limitation to new trials. When considering cell therapy, scientists, physicians and regulators must keep in mind that ATMP must be affordable for patients and society. Interestingly, scientists are largely responsible for current guidelines, and should revisit those recommendations based on factors such as cost (55, 132).

## Immunomonitoring of Tolerance-Inducing Cellular Therapies

### Tackling Immunomonitoring in Tolerogenic Therapies

Since cell-based therapies are becoming more common, it is important to reliably monitor the immune system for both desired and undesired immunological effects. Rigorous immunomonitoring will therefore provide information about the safety of these treatments, ideally at early time points after CTT administration. In addition it will give insight in the therapy-related mechanisms of tolerance-induction and maintenance and may aid in patient-tailoring of therapy. To accurately measure the effects, especially across different trials, it will require introduction of harmonized and validated immunomonitoring assays.

There are a number of possible assays that can determine cell therapy effects in humans. For instance, measurement of circulating cytokines, C-reactive protein or changes in antibody titer can determine immune status. Similarly, measuring CD4^+^ T cell responses after viral-antigen stimulation is possible by flow cytometry via CD40L expression or cytokine production in a functional assay; these assays could potentially identify non-specific immunosuppressive effects (133). Although the completed clinical trials have shown so far that cell-based tolerogenic therapies are safe and do not cause serious undesirable immune responses (96, 99, 103, 104), the extent to which these treatments achieve therapeutic efficacy remains largely undetermined. Current cell-based tolerogenic trials in autoimmunity and transplantation include clinical outcome measures such as C-peptide response, insulin consumption or reduction of immunosuppressive doses. However, clinical endpoints may not necessary reflect the efficacy of cell-based therapies, since the tolerogenic effect of the transferred cells may not directly lead to immediate changes in systemic parameters such as inflammation, potentially underestimating a longer term effect. It is therefore important that future clinical trials incorporate suitable monitoring methods to assess the immunomodulatory effects of cellular therapies.

### General vs. Specific Monitoring Assays

To assess therapeutic effectiveness, different methods have been proposed to identify tolerogenic responses. The assessment of *in vitro* autoreactive or donor-specific T cells responses prior to and after treatment could provide a precise evaluation of therapeutic efficacy. Antigen-specific assays allow discrimination between targeted tolerance to the induction of general immune suppression and loss of responses to pathogens. In addition, these methods provide an efficacy readout for antigen-specific therapies such as tolerogenic APC loaded with antigens or the generation of donor-specific Treg. The identification of antigen-specific immune responses by ELISPOT (134) or through flow cytometry detection of CD40L upregulation (135) have shown promising results in predicting kidney and liver allograft rejection, suggesting potential applicability for the evaluation of tolerogenic therapies. Monitoring targets will be different depending on the main immune population involved in the disease (e.g., CD4, CD8 T cells or antibodies). Unfortunately, the antigens mediating the immune responses in autoimmunity are not always available or identified (as discussed in paragraph Antigen Specificity of tolAPC-Based Immunomodulation); HLA antigens in the case of transplantation are known and can be used, or stimulation with donor or donor-matched cells is possible. Though less specific, identification of phenotypic or functional changes by flow cytometry on the total pool of cells targeted (e.g., Treg) by the tolerogenic treatment may reveal therapeutic effectiveness. Indeed, the acquisition of tolerance in animal models and in the clinic is associated with an increased number of regulatory cells and decreased pro-inflammatory function of innate and adaptive immune cells (5, 136, 137). Therefore, flow cytometry analysis to delineate the distribution and activation status of different cell types, or *in vitro* assays to evaluate the suppressive and inflammatory function of circulating cells can provide a non-specific approach to assess the development of tolerogenic properties (138). Other non-specific strategies such as gene expression profiling of circulating immune cells or tissue biopsies can add to the functional assessment of immune responses. Furthermore, there is a growing body of work focusing on the validation of transcriptional signatures to predict transplantation tolerance in liver and kidney transplant recipients (139–141), which may prove to be a valuable tool in assessing the efficacy of tolerogenic therapies.

### Tracking of Cellular Product

The evaluation of homeostatic characteristics of infused cells such as survival, stability or tissue migration, constitutes another monitoring objective to assess therapeutic efficacy. Being able to track infused cells will help to determine the best site for administration/application of tolerogenic cell products. While simple phenotypic detection (flow cytometry) of the transferred cell subset after treatment suggests the presence of infused cells, it does not distinguish transferred from endogenous cells. Therefore, current techniques for tracking infused cells depend on direct cell labeling strategies, such as indium labeling or deuterium introduction during *ex vivo* expansion, with subsequent isotope detection in the different tissues or compartments (99, 103, 142). Unfortunately, this method is only semi-quantitative, since individual cells are not detected. Individual cells can be labeled with rare earth metals and detected with precision *in vivo* using laser ablation-inductively coupled plasma-mass spectrometry, but so far this has only been tested in mouse models (143, 144). Other emerging therapeutic approaches such as CAR-Treg could take advantage of genetic modifications to adapt reported gene imaging strategies to detect the transferred cells by non-invasive methods (e.g., MRI, PET, SPECT) (145, 146). Additionally, the use of T cell receptor (TCR) engineered Treg or *ex vivo* expanded antigen-specific Treg create the opportunity to track infused cells by predefining the TCR clones and identifying them among the T cell compartment repertoire through TCR sequencing analysis (147).

### Harmonization to Allow Comparison

Current tolerogenic cell-based trials are highly heterogeneous, comprising different cell types with variable manufacturing approaches, and targeting various autoimmune diseases and transplantation settings. Furthermore, by their very nature such trials tend to involve low numbers of patients, often participating in different centers or countries. It is therefore essential to establish common immunomonitoring strategies in the research community to achieve robust and reliable data which can be compared and combined between trials. First, not all the immunomonitoring methods are similarly standardisable, and second, not all the centers have the technical expertise or infrastructure to perform certain assays. Sample collection and storage of whole blood or tissue biopsies for transcriptional analysis does not involve extensive sample manipulation, making standardization of this method achievable. On the contrary, while flow cytometry analysis of circulating blood is an accessible technology, instrumentation must be calibrated appropriately to accurately define cell subsets, and analysis of the data needs to be strictly regulated. Although centralized phenotypic analysis in multicenter trials is feasible (105), this involves significant logistical challenges, including the decision to either test fresh or frozen samples; frozen samples sacrifice accuracy due to loss of certain cell populations during separation and freezing procedures. In general, flow cytometry standardization requires extensive cooperation between centers and precise planning. To further harmonize flow cytometry data implementation of automated gating approaches will be of outmost importance in the future (148–150). Finally, functional *in vitro* assays also represent a challenging method to standardize, involving different approaches depending of the cell type and function. Nevertheless, several efforts have been made to establish a minimal harmonization of antigen-specific functional assays (56). While these assays are likely to be the most informative in the assessment of therapeutic efficacy, it is unlikely that current assay results will be directly comparable between independent trials. Therefore, the inclusion of adequate control cohorts and reference groups in the assays, considering the specific cell therapy approaches and disease characteristics, remains an objective to achieve feasible comparisons (107).

## Final conclusion

Tolerance-inducing cellular therapies have great potential. Several cell types are now in early-stage clinical trials, including various types of Treg and tolAPC (including tolDC and Mreg) (Tables 1, 2). At the present time it is unclear which of these cell types will prove most suitable as a cell-based therapy; each likely has particular advantages that may be suitable for one particular disease or another. In Table 3, the main specific limitations to be considered for the treatment of autoimmunity or transplantation with tolerance-inducing cell therapies are summarized. It becomes clear that although we might treat these diseases with the same manufactured cell product, the optimal treatment regime could be quite different.

**Table 3:** Main specific differences in tolerance-inducing cell treatment between transplantation and autoimmune disease setting.

	**Transplantation**	**Autoimmunity**
Antigen	- Alloantigens (MHC alleles and other disparities) - Autoantigens in case of underlying or de novo developed autoimmune diseases	- Autoantigen not always known - Epitope spreading might occur during disease progression
Pathogenic immune response	- Normal, but undesired, immune response against foreign antigen	- Loss of tolerance to self-antigen
Timing	- Time point of antigen contact is known, treatment can be given around time point of transplantation	- Disease already develops before clinical symptoms - Better diagnosis and biomarkers are needed to be able to intervene at earlier time point
Route of administration	- Intravenous	- Intradermal - Local injection in affected tissue or draining lymph node of the tissue is to be considered
Co-medication	- High dose of conventional immune suppression (steroids, CNI, MMF) +/- antibody-based induction therapy at the moment of transplantation	- Varies and is disease specific
Clinical efficacy evaluation	- Prevention of acute rejections - Reduction conventional immune suppression. It is difficult to lower co-medication without good markers to predict transplant tolerance	- Disease-dependent. E.g. In T1D C-peptide response or insulin consumption can be determined. In other AID disease-specific scores can be used. - Depending on disease progression on moment of application. Irreversible tissues destruction will not improve. New relapses/lesions can be scored.
Immunomonitoring	- Donor antigen-specific T cells - Donor specific antibodies	- Autoantigen not always known. When known, T cell responses are difficult to detect, since they are very low frequent and of low affinity

Immunomonitoring is an indispensable aspect of current and future tolerogenic cell-based therapies that will provide fundamental information to understand and optimize cell therapies. Monitoring will also aid in identifying biomarkers with the capacity for early identification of therapy responders and non-responders and patient-tailoring of therapy. As part of the overall strategy to increase implementation of ATMPs, it will be critical to harmonize GMP manufacturing protocols, product characterization and immunomonitoring. Minimal information models such as MITAP and MiTREG (132, 151) will serve as important tools in this respect.

## Author Contributions

All authors listed have made a substantial, direct and intellectual contribution to the work, and approved it for publication.

### Conflict of Interest Statement

PT Medical University of Gdańsk–owns a patent related to presented content. The remaining authors declare that the research was conducted in the absence of any commercial or financial relationships that could be construed as a potential conflict of interest.
